# Identification of novel differentially expressed genes in type 1 diabetes mellitus complications using transcriptomic profiling of UAE patients: a multicenter study

**DOI:** 10.1038/s41598-022-18997-w

**Published:** 2022-09-29

**Authors:** Bashair M. Mussa, Thenmozhi Venkatachalam, Ankita Srivastava, Abeer Al-Habshi, Elamin Abdelgadir, Alaaeldin Bashier, Fatheya Al Awadi, Khadija Hafidh, Rifat Hamoudi, Salah Abusnana

**Affiliations:** 1 Basic Medical Science Department, College of Medicine, University of Sharjah, P.O. Box: 27272, Sharjah, United Arab Emirates; 2 Department of Physiology and Immunology, College of Medicine, Khalifa University, Abu Dhabi, United Arab Emirates; 3 Sharjah Institute for Medical Research, University of Sharjah, Sharjah, United Arab Emirates; 4 Endocrinology Department, Dubai Hospital, Dubai, United Arab Emirates; 5 Internal Medicine Department, Rashid Hospital, Dubai, United Arab Emirates; 6 Clinical Science Department, College of Medicine, University of Sharjah, Sharjah, United Arab Emirates; 7 Division of Surgery and Interventional Science, University College London, London, UK; 8 Diabetes and Endocrinology Department, University Hospital Sharjah, Sharjah, United Arab Emirates

**Keywords:** Diseases, Endocrinology

## Abstract

Type 1 diabetes mellitus (T1DM) is a chronic metabolic disorder that mainly affects children and young adults. It is associated with debilitating and long-life complications. Therefore, understanding the factors that lead to the onset and development of these complications is crucial. To our knowledge this is the first study that attempts to identify the common differentially expressed genes (DEGs) in T1DM complications using whole transcriptomic profiling in United Arab Emirates (UAE) patients. The present multicenter study was conducted in different hospitals in UAE including University Hospital Sharjah, Dubai Hospital and Rashid Hospital. A total of fifty-eight Emirati participants aged above 18 years and with a BMI < 25 kg/m^2^ were recruited and forty-five of these participants had a confirmed diagnosis of T1DM. Five groups of complications associated with the latter were identified including hyperlipidemia, neuropathy, ketoacidosis, hypothyroidism and polycystic ovary syndrome (PCOS). A comprehensive whole transcriptomic analysis using NGS was conducted. The outcomes of the study revealed the common DEGs between T1DM without complications and T1DM with different complications. The results revealed seven common candidate DEGs, *SPINK9*, *TRDN*, *PVRL4*, *MYO3A*, *PDLIM1*, *KIAA1614* and *GRP* were upregulated in T1DM complications with significant increase in expression of *SPINK9* (Fold change: 5.28, 3.79, 5.20, 3.79, 5.20) and *MYO3A* (Fold change: 4.14, 6.11, 2.60, 4.33, 4.49) in hyperlipidemia, neuropathy, ketoacidosis, hypothyroidism and PCOS, respectively. In addition, functional pathways of ion transport, mineral absorption and cytosolic calcium concentration were involved in regulation of candidate upregulated genes related to neuropathy, ketoacidosis and PCOS, respectively. The findings of this study represent a novel reference warranting further studies to shed light on the causative genetic factors that are involved in the onset and development of T1DM complications.

## Introduction

Globally, there is a dramatic increase in both the prevalence and incidence of type 1 diabetes mellitus (T1DM) with a current estimate of 1.1 million children and adolescents (< 20 years) living with T1DM around the world^1^. Given the age of the T1DM patients, these statistics will have a significant impact in shaping the future of public health and associated economic burden^2^.

Genetic heterogeneity of T1DM has received much attention recently as it provides new insights into the pathogenesis and development of various complications that are associated with T1DM^3^. Although the evidence of this heterogeneity is growing fast, the interpretation and the clinical implications are yet to be determined.

Earlier genomic studies have identified HLA and non-HLA loci on chromosome 6 that are involved in expression of T1DM complications^4^. In addition, large-scale studies of 8114 T1DM patients among 6707 families in an American cohort have strongly suggested genetic basis for susceptibility to T1DM microvascular complications (MVCs) including retinopathy, nephropathy, and neuropathy^5^. Moreover, studies in Finnish population have identified genes that predispose patients with T1DM to develop advanced diabetic nephropathy^6^.

A more recent study of 415 families investigated T1DM-related MVCs and tested the hypothesis that T1DM associated with MVCs is genetically distinct from T1DM without MVCs^3^. Interestingly, the findings showed that the latter exhibited distinct genetic signature compared to the T1DM patients with MVCs^3^. In agreement with these findings, the genome-wide association studies have identified more than 60 susceptibility regions within the human genome for T1DM which are evidenced by single nucleotide polymorphisms^7^.

Another serious complication of T1DM is diabetic ketoacidosis (DKA), which is one of the most common causes of morbidity and mortality in T1DM^8^. A Finnish study has investigated the risk of developing DKA in T1DM and whether it is related to HLA genotypes and the outcomes have shown that children with high to moderate risk of T1DM HLA-DQB1 genotypes had lower frequency of DKA than children with other genotypes^9^. On the other hand, an association has been reported between high chance of presenting DKA and HLA-associated high-risk genotypes using HLA DQA1-DQB1 haplotype combination genotypes^10^. Interestingly, it was also found that having at least one first-degree relative with history of T1DM decreased the risk of DKA regardless of HLA genotype^10^.

In addition, earlier studies which investigated the role of dietary fats in development of hyperlipidemia in T1DM have shown that abnormal lipid profile was common in T1DM children and adolescents who were well-adherent to healthy diet suggesting that other factors including genetics are involved in pathogenesis of hyperlipidemia as a complication that is associated with T1DM^11^. More recent reports have documented that heterozygous mutations in genes encoding lipoprotein lipase are associated with hypertriglyceridemia in newly diagnosed T1DM^12^.

Moreover, there are other prevalent endocrine complications associated with T1DM including polycystic ovary syndrome (PCOS) and thyroid dysfunction which produce negative consequences on the quality of life of T1DM patients^13,14^. Although genetic predisposition has been suggested as a prerequisite for development of these complications, more studies are required to elucidate the implication of the genetic factors.

In the present study a comprehensive transcriptomic analysis was conducted in the United Arab Emirates (UAE, Emirati) patients with T1DM and multiple complications to better our understanding of the candidate genes that are involved in the pathogenesis of these complications. To the best of our knowledge this is the first study to investigate the candidate genes that are associated with different complications of T1DM in Emirati patients.

The aim of the present study is to determine the differentially expressed genes (DEGs) that are common between T1DM complications including hyperlipidemia, diabetes neuropathy, PCOS, DKA and hypothyroidism using transcriptomic profiling.

## Methods

### Study population

The present study is a cross sectional study which included a total of fifty-eight participants of UAE nationality (Emirati) aged above 18 years and with a BMI < 25 kg/m^2^. Forty-five of these participants had a confirmed diagnosis of T1DM and were recruited from University Hospital Sharjah (UHS), Dubai Hospital (DH) and Rashid Hospital (RH). Exclusion criteria were (i) patients with type 2 DM, (ii) non-Emirati patients, (iii) BMI > 25 kg/m^2^, (iv) patients with chronic kidney disease and (v) severe liver disease. The study was approved by the ethics committee of University of Sharjah (UOS, REC-17-08-08-01), UHS (UHS-09042018), DH (DSREC-09/2018-13) and RH (DSREC-07/2019-05) and conducted in accordance with the Declaration of Helsinki. All participants were asked to sign an informed-consent form written in their native language. This form was approved by the ethics committees prior to the onset of the recruitment process. Different groups were established based on the absence or presence of T1DM complications as show in Table 1. The diagnosis of each complication has already been confirmed and documented by consultant diabetologists and endocrinologists in the medical records of the patients and this information was used to create different complication groups.

**Table 1:** Groups of the studied population.

Group	Subjects number
Control	13
T1DM without complications	15
T1DM with hyperlipidemia	11
T1DM with neuropathy	7
T1DM with ketoacidosis	6
T1DM with hypothyroidism	6
T1DM with PCOS	5

Total number of subjects in each group has been presented including control group, T1DM without complications, T1DM with hyperlipidemia, neuropathy, ketoacidosis, hypothyroidism and PCOS.

*T1DM* Type I Diabetes Mellitus, *PCOS* Polycystic Ovary Syndrome.

### Data collection

Demographic and baseline clinical data for the participants were collected from the electronic medical record systems of the three hospitals: UHS, DH, and RH. This data included age (years), gender, diabetes duration (years) and age at diagnosis (years). In addition, more data were collected about anthropometric measurements which included: height (cms), weight (kgs) and BMI (kg/m^2^). In addition, vital signs were also collected including systolic blood pressure (mmHg), diastolic blood pressure (mmHg) and heart rate (beats/min).

### Blood sample collection

From each subject, about 8 ml of blood sample was collected once and the following tests were conducted for the laboratory parameters of kidney and liver function: haemoglobin A1c (HbA1c, %), fasting blood glucose, insulin levels (mU/L), total cholesterol (mmol/L), triglycerides (mmol/L), high-density lipoprotein (HDL, mmol/L), low-density lipoprotein (LDL, mmol/L), c-reactive protein (mg/L), creatinine (mmol/L) and C-peptide (nmol/L). For genetic and transcriptomic analysis, RNA was extracted from the same blood samples using the Qiazol® method (Qiagen, Hilden, Germany) as per the manufacturer’s instructions.

### Whole transcriptome sequencing

RNA samples extracted from patients were used to carry out whole transcriptomic analysis using AmpliSeq whole Transcriptome kit on S5 XL System. In brief, ~ 30 ng of Turbo DNase treated RNA was used to synthesize cDNA using SuperScript VILO cDNA Synthesis kit (Invitrogen, Carlsbad, USA) followed by amplification with Ion Ampliseq gene expression core panel primers. Enzymatic shearing was performed using FuPa reagent to obtain amplicons of ~ 200 bp and the sheared amplicons were ligated with the adapter and unique barcodes. After that, the prepared library was purified using Agencourt AMPure XP beads (Beckman Coulter, Brea, USA) to get rid of any unamplified amplicons and adapter dimers and the purified library was quantified using Ion Taqman library quantitation kit (Applied Biosystems, Foster City, USA). The libraries were further diluted to 100 pM and pooled equally with four individual samples per pool. The pooled libraries were amplified using emulsion PCR on Ion One Touch2 instruments (OT2) and enrichment was done on Ion One Touch ES following manufacturer’s instruction. Prepared template libraries were then sequenced on Ion S5 XL Semiconductor sequencer using Ion 540 Chip. All reagents used for whole transcriptome sequencing and analysis were purchased from Thermo Fisher Scientific, Waltham, USA, unless mentioned otherwise.

### Bioinformatics analysis

RNA-seq data were analyzed using Ion Torrent Software Suite version 5.4. Alignment was carried out using the Torrent Mapping Alignment Program (TMAP). TMAP is optimized for aligning the raw sequencing reads against reference sequence derived from hg19 (GRCh37) assembly and the specificity and sensitivity was maintained by implementing a two-stage mapping approach by employing BWA-short^15^, BWA-long^16^, SSAHA^17^, Super-maximal Exact Matching^18^ and Smith-Waterman algorithm^19^ for optimal mapping. Raw read counts of the targeted genes were performed using samtools (samtools view –c –F 4 –L bed_file bam_file) and the number of expressed transcripts was confirmed after Fragments Per Kilobase Million (FPKM) normalization. DEGs analysis was performed using R/Bioconductor package DESeq2 with raw read counts from RNASeq and AmpliSeq. Read count normalization was performed using DESeq2 (Ref: https://genomebiology.biomedcentral.com/articles/10.1186/s13059-014-0550-8). Genes with less than ten normalized read counts were excluded from further analysis.

### Statistical analysis

All data is expressed as mean (± SD). Correlation of clinical data with genomics data was carried out using multivariate analysis. ANOVA was used with Bonferroni multiple testing correction to identify significant genetic and clinical variables. All statistical analyses were conducted using SPSS software (version 24). P < 0.05 is statistically significant.

## Results

### Baseline demographic and clinical characteristics of the studied groups

The present study included young, lean Emirati participants and seven groups were established including Group 1 (Control, healthy participants, n = 13), Group 2 (T1DM patients without complications, n = 15) and Group 3 to Group 7 which include patients with various T1DM complications (Table 1). The details of these groups are as follows: Group 3 (T1DM with hyperlipidemia, n = 11), Group 4 (T1DM with neuropathy, n = 7), Group 5 (T1DM with ketoacidosis, n = 6), Group 6 (T1DM with hypothyroidism, n = 6) and Group 7 (T1DM with PCOS, n = 5) (Table 1). The average age of the participants across Group 1 to Group 7 was 27.38 ± 10.67, 24.26 ± 6.02, 32.81 ± 8.48, 32.57 ± 8.10, 27.16 ± 6.49, 29.16 ± 7.13 and 22.8 ± 3.83, respectively (Table 2). As shown in Table 2, all the participants had normal weight with BMI ranges between 21.71 ± 5.41 kg/m^2^ and 24.11 ± 3.12 kg/m^2^. In addition, Group 1 had normal range of HbA1c (5.34 ± 0.49%) which is significantly lower than all T1DM groups and a similar pattern was observed in the lipid profile of Group 1 (Cholesterol, 66.82 ± 78.10 mg/dL; Triglyceride, 21.48 ± 26.33 mg/dL; HDL, 20.47 ± 28.41 mg/dL; LDL, 34.48 ± 42.12 mg/dL).

**Table 2:** Baseline demographic and clinical characteristics of the studied population: control, T1DM without complications and T1DM with complications.

Variables	Control	T1DM (without complications)	T1DM (hyperlipidemia)	T1DM (neuropathy)	T1DM (ketoacidosis)	T1DM (hypothyroidism)	T1DM (PCOS)
Subjects (n)	13	15	11	7	6	6	5
Age (years)	27.38 ± 10.67	24.26 ± 6.02	32.81 ± 8.48	32.57 ± 8.10	27.16 ± 6.49	29.16 ± 7.13	22.8 ± 3.83
Gender (female), n (%)	12 (92.3%)	8 (53.33%)	5 (45.45%)	5 (71.42%)	5 (83.33%)	4 (66.66%)	5 (100%)
Gender (male), n (%)	1 (7.69%)	7 (46.66%)	6 (54.54%)	2 (28.51%)	1 (16.66%)	2 (33.33%)	0 (0%)
Age at diagnosis (year)		13.33 ± 5.75	14.72 ± 6.23	18 ± 12.78	16.33 ± 4.08	11.33 ± 5.24	13 ± 5.04
Diabetes duration (years)		10.93 ± 7.93	18.09 ± 6.65	17 ± 7.09	10.83 ± 5.60	17.83 ± 7.11	9.8 ± 3.03
Height (cm)	161.11 ± 5.95	163.7 ± 7.34	163.95 ± 8.60	166.14 ± 13.43	163.75 ± 4.83	162.33 ± 7.42	160.8 ± 3.75
Weight (kg)	56.78 ± 16.01	60 ± 7.63	64.14 ± 11.42	67.5 ± 16.58	59.81 ± 3.87	57.55 ± 9.87	56.38 ± 9.34
BMI (kg/m^2^)	21.71 ± 5.41	22.48 ± 3.19	23.70 ± 3.00	24.11 ± 3.12	22.31 ± 1.06	21.96 ± 4.22	21.79 ± 3.07
HbA1c (%)	5.34 ± 0.49	8.04 ± 1.14	8.21 ± 1.88	8.62 ± 2.05	8.35 ± 1.76	8.66 ± 2.05	8.52 ± 1.79
Cholesterol (mg/dL)	66.82 ± 78.10	125.87 ± 57.05	159.60 ± 96.80	141.63 ± 111.08	152.8 ± 32.29	141.81 ± 108.83	139 ± 23.51
Triglyceride (mg/dL)	21.48 ± 26.33	54.64 ± 31.48	106.64 ± 104.88	116.02 ± 130.67	59.8 ± 18.67	103.96 ± 117.66	63.2 ± 22.01
HDL (mg/dL)	20.47 ± 28.41	44.73 ± 22.56	42.11 ± 25.07	35.85 ± 26.65	60.4 ± 10.87	43.78 ± 33.25	53.4 ± 3.84
LDL (mg/dL)	34.48 ± 42.12	78.59 ± 37.89	101.34 ± 70.49	83.61 ± 63.07	91 ± 29.60	84.71 ± 63.29	85.2 ± 25.69

Control Group includes healthy subjects without diabetes; T1DM Group includes patients with T1DM without complications. Complication Groups include patients with T1DM complications (Hyperlipidemia, Neuropathy, Ketoacidosis, Hypothyroidism, PCOS).

*T1DM * Type 1 Diabetes Mellitus, *PCOS* Polycystic Ovarian Syndrome, *BMI* Body Mass Index, *HDL* High-Density Lipoprotein, *LDL* Low Density-Lipoprotein.

### Comparison of gene expression profiles of different complications of T1DM

**Figure 1:**
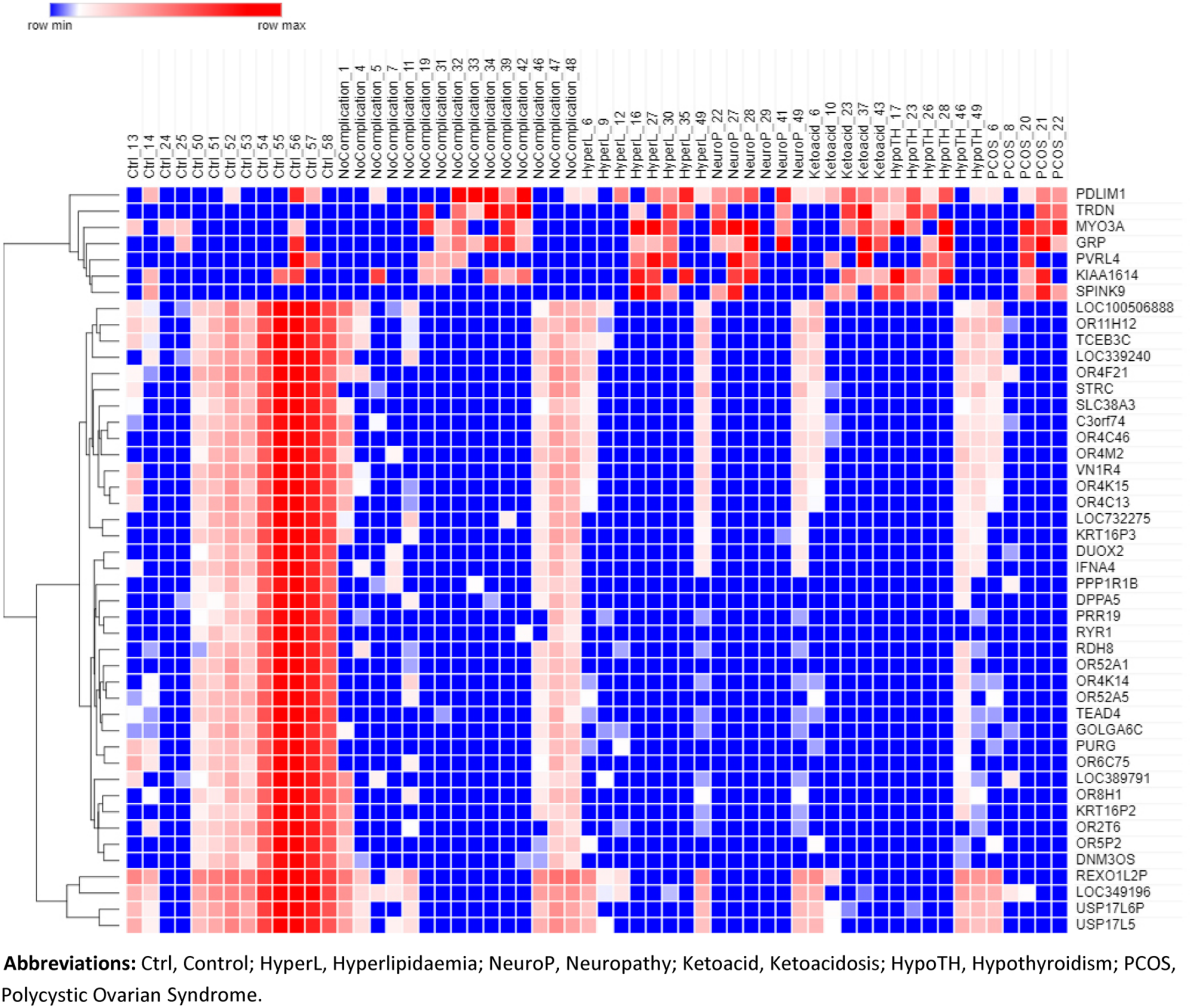
Heat map of overlapping differentially expressed genes as identified by ANOVA and eBayes analysis of healthy controls, T1DM with no complications and T1DM with complication groups. Red color represents upregulated genes whereas blue color represents downregulated genes. Color scale is shown on the top left corner of the figure. *Ctrl* control, *HyperL* Hyperlipidemia, *NeuroP* Neuropathy, *Ketoacid* Ketoacidosis, *HypoTH* Hypothyroidism, *PCOS* Polycystic Ovarian Syndrome.

RNA-seq analysis of seven groups including control, T1DM without complication (DWC), hyperlipidemia, neuropathy, ketoacidosis, hypothyroidism and PCOS showed that T1DM complications share a common group of DEGs with DWC group. In addition, all the complications share seven common upregulated genes and thirty-nine downregulated gene. Clustering analysis of these upregulated and downregulated genes are shown in Heatmap in Fig. 1.

The transcriptomic analysis had shown that each T1DM complication exhibited a distinct group of DEGs with log2 fold change > 2. As shown in Table 3, the total number of upregulated genes was 914 (hyperlipidemia), 1571 (neuropathy), 746 (ketoacidosis), 1160 (hypothyroidism) and 359 (PCOS) and the total number of down regulated genes for the same T1DM complications was 2623, 2429, 2678, 2598, 2946, respectively (Table 3).

**Table 3:** Total number of upregulated and downregulated genes per complication group with log2 fold change greater than 2.

Complications	Upregulated Genes (log2 fold change > 2)^a^	Downregulated Genes (log2 fold change > 2)^b^
Hyperlipidemia	914	2623
Neuropathy	1571	2429
Ketoacidosis	746	2678
Hypothyroidism	1160	2598
PCOS	359	2946

^a^Indicates the total number of differentially expressed genes (Upregulated) and ^b^indicates total number of differentially expressed genes (Downregulated) across five diabetes complications (Hyperlipidemia, Neuropathy, Ketoacidosis, Hypothyroidism and PCOS).

*PCOS* Polycystic Ovarian Syndrome.

**Figure 2:**
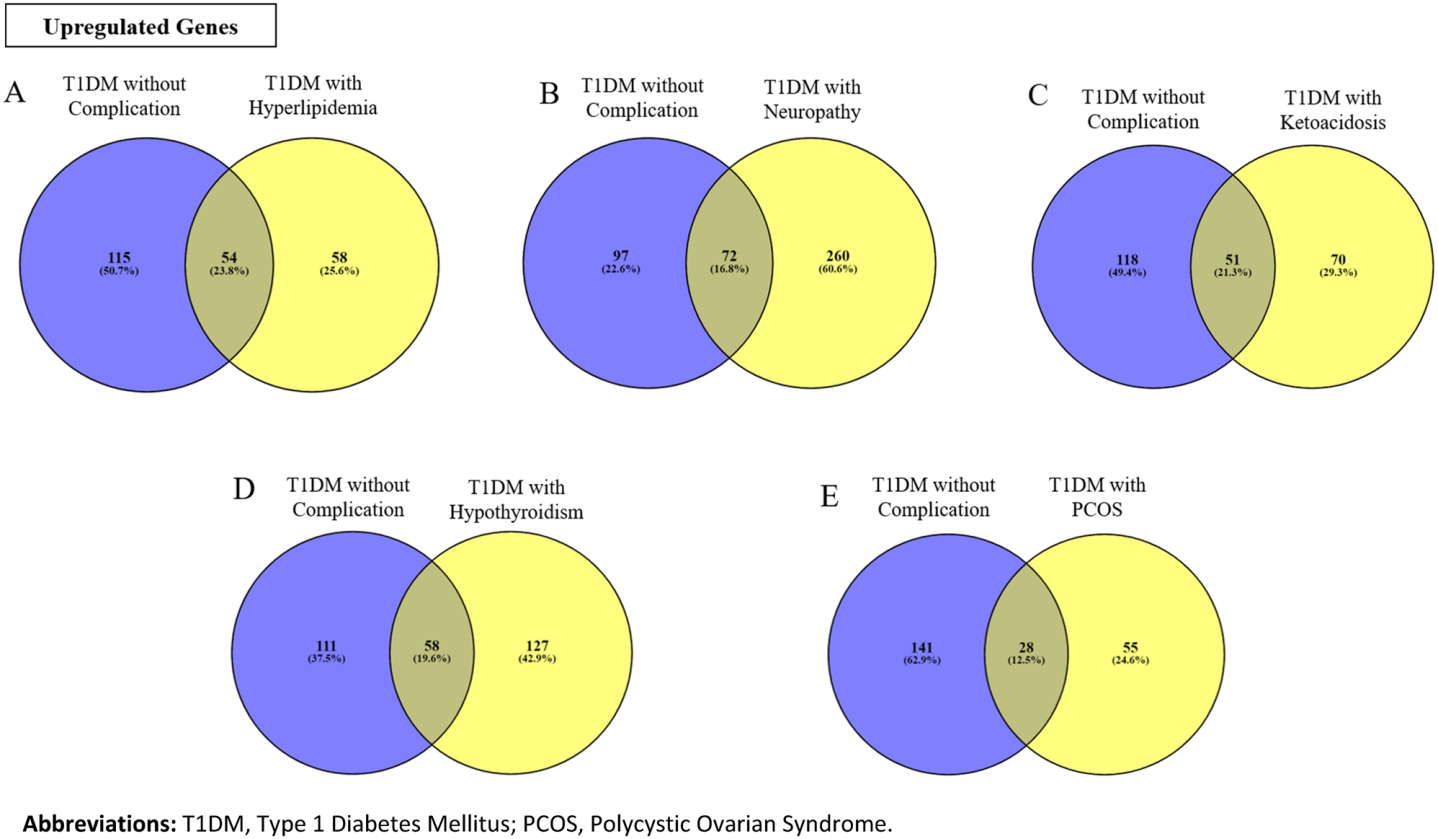
Upregulated and common DEGs between the T1DM without complications (blue circles) and T1DM with complication groups (yellow circles; (**A**) Hyperlipidemia, (**B**) Neuropathy, (**C**) Ketoacidosis, (**D**) Hypothyroidism and (**E**) Polycystic Ovarian Syndrome [PCOS]). Overlap between the two circles denotes the number and corresponding percentage of the common genes which was highest between T1DM without complications vs T1DM with hyperlipidemia (23.8%) and lowest for the PCOS group (12.5%).

As shown in Fig. 2, common DEGs between DWC and T1DM with different complications have been identified and this includes 54, 72, 51, 58 and 28 common upregulated genes between DWC and hyperlipidemia, neuropathy, ketoacidosis, hypothyroidism and PCOS, respectively. The list of these DEGs is provided in Supplementary Tables 1 to 5.

### Functional annotation and enrichment analysis of upregulated DEGs

**Figure 3:**
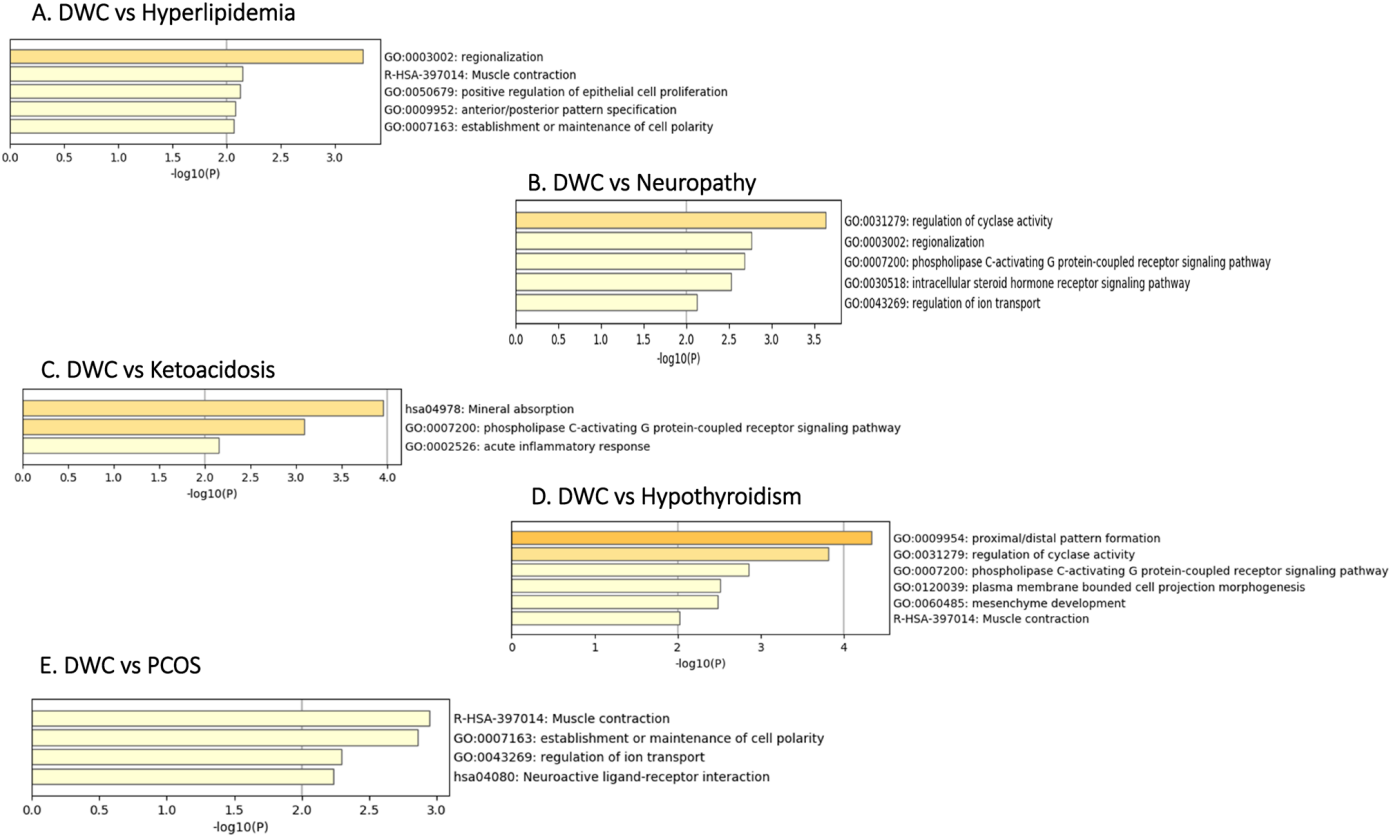
Enriched pathways and functional clusters of upregulated common genes between DWC vs T1DM with complication groups ((**A**) Hyperlipidemia, (**B**) Neuropathy, (**C**) Ketoacidosis, (**D**) Hypothyroidism and (**E**) Polycystic Ovarian Syndrome [PCOS]).

Top functional pathways were identified for the common upregulated genes (Fig. 3). The top five pathways for the common upregulated genes between DWC and hyperlipidemia were regionalization, muscle contraction, positive regulation of epithelial cell proliferation, anterior/posterior pattern specification and establishment or maintenance of cell polarity (Fig. 3A). In addition, the top five functional pathways that were associated with the common upregulated genes between DWC and neuropathy included regulation of cyclase activity, regionalization, phospholipase c-activating G protein-coupled receptor signaling, intercellular steroid hormones receptor signaling pathway and regulation of ion transport (Fig. 3B). The top three functional pathways for the common upregulated genes between DWC and ketoacidosis were mineral absorption, phospholipase c-activating G protein-coupled receptor signaling pathway and acute inflammatory response (Fig. 3C). Furthermore, there were six top functional pathways related to the common upregulated genes between DWC and hypothyroidism and this included proximal and distal pattern formation, regulation of cyclase activity, phospholipase c-activating G protein-coupled receptor signaling pathway, mesenchyme development and muscle contraction (Fig. 3D). For the common upregulated genes between DWC and PCOS, four top functional pathways were identified including muscle contraction, establishment and maintenance of cell polarity, regulation of ion transport and neuroactive ligand-receptor interaction (Fig. 3E).

### Functional annotation and enrichment analysis of downregulated DEGs

**Figure 4:**
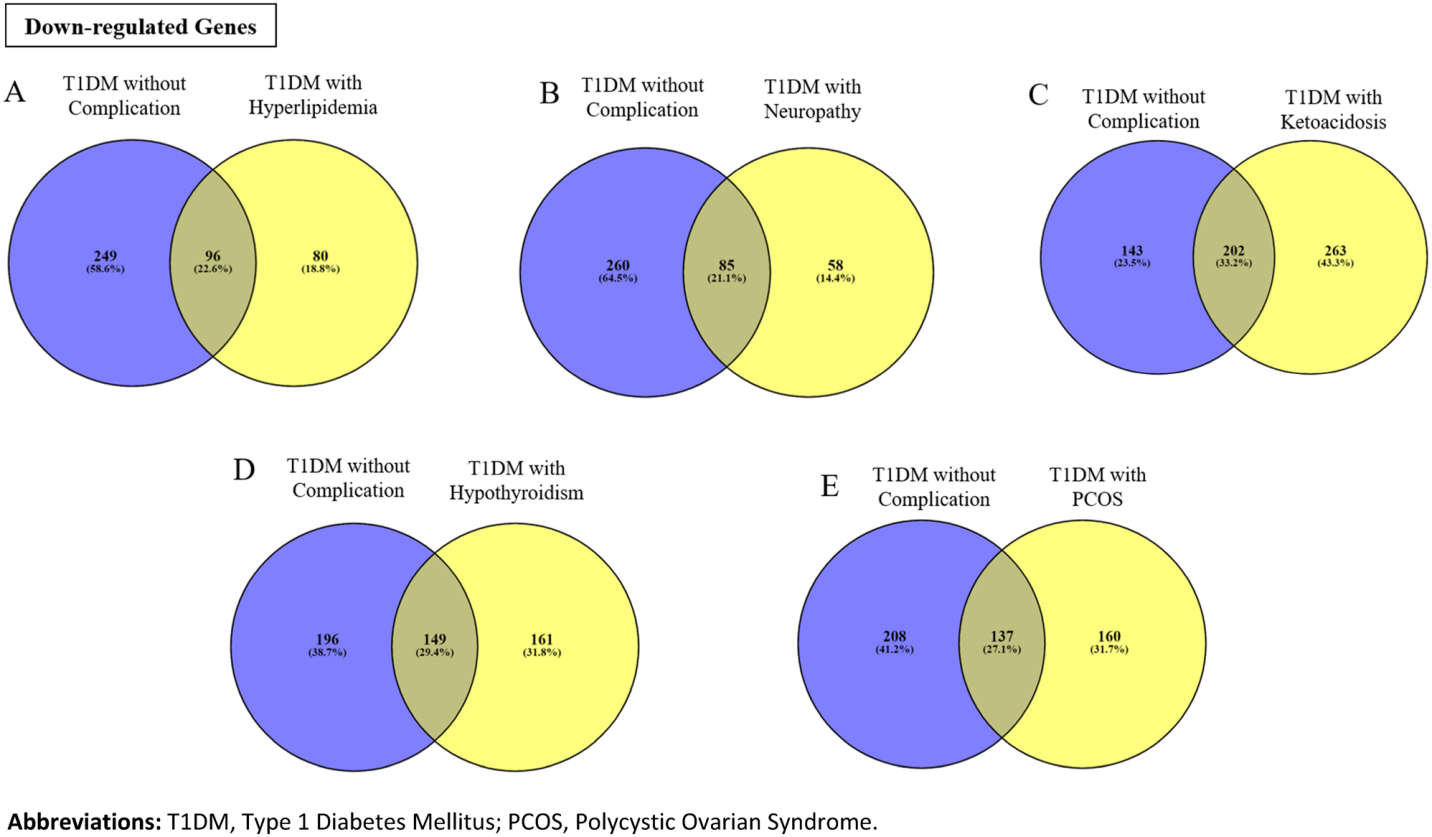
Downregulated and common DEGs between the T1DM without complications (blue circles) and T1DM with complication groups (yellow circles); (**A**) Hyperlipidemia, (**B**) Neuropathy, (**C**) Ketoacidosis, (**D**) Hypothyroidism and (**E**) Polycystic Ovarian Syndrome [PCOS]). Overlap between the two circles denotes the number and corresponding percentage of the common genes which was highest between T1DM without complications vs T1DM with ketoacidosis (33.2%) and lowest for the neuropathy group (21.1%).

As shown in Fig. 4, common downregulated DEGs between DWC and T1DM with different complications have been identified and this includes 96, 85, 202, 149 and 137 common downregulated genes between DWC and hyperlipidemia, neuropathy, ketoacidosis, hypothyroidism and PCOS, respectively. The list of these is provided in Supplementary Tables 6 to 10.

**Figure 5:**
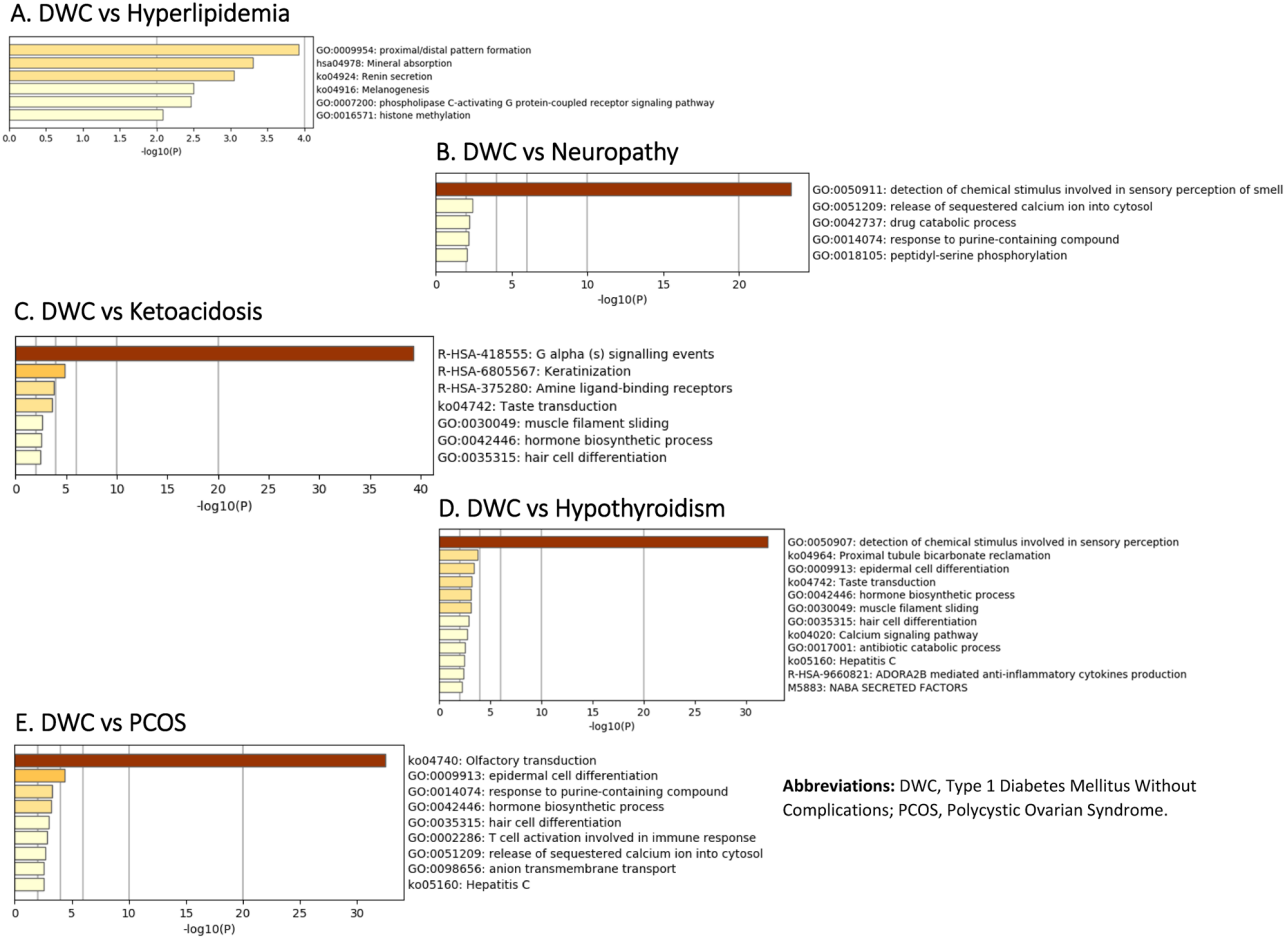
Enriched pathways and functional clusters of downregulated common genes between DWC vs T1DM with complication groups ((**A**) Hyperlipidemia, (**B**) Neuropathy, (**C**) Ketoacidosis, (**D**) Hypothyroidism and (**E**) Polycystic Ovarian Syndrome [PCOS]).

Top functional pathways were identified for these common downregulated genes (Fig. 5). The top six pathways for the common downregulated genes between DWC and hyperlipidemia were proximal/distal pattern formation, mineral absorption, renin secretion melanogenesis, phospholipase c-activating G protein-coupled receptor signaling pathway and histone methylation (Fig. 5A).

Common downregulated genes between DWC and neuropathy were associated with top five pathways including detection of chemical stimulus involved in sensory perception of smell, release of sequestered calcium ion cytosol, drug catabolic process, response to purine-containing compound and peptide-serine phosphorylation (Fig. 5B). More functional pathways were associated with the common downregulated genes between DWC and ketoacidosis, and this includes G alpha signaling events, keratinization, amine ligand-binding, taste transduction, muscle filament sliding, hormone biosynthesis process and hair cell differentiation (Fig. 5C). Twelve functional pathways were identified in association with the common downregulated genes between DWC and hypothyroidism and the top six of the twelve included detection of chemical stimulus involved in sensory perception, proximal tubule bicarbonate reclamation, epidermal cell differentiation, taste transduction, hormone biosynthesis process and muscle filament sliding (Fig. 5D). In addition, another nine pathways were associated with the common downregulated genes between DWC and PCOS, the top six of these nine pathways were olfactory transduction, epidermal cell differentiation, response to purine-containing compound, hormone biosynthesis process, hair cell differentiation and T-cell activation involved in immune response (Fig. 5E).

**Figure 6:**
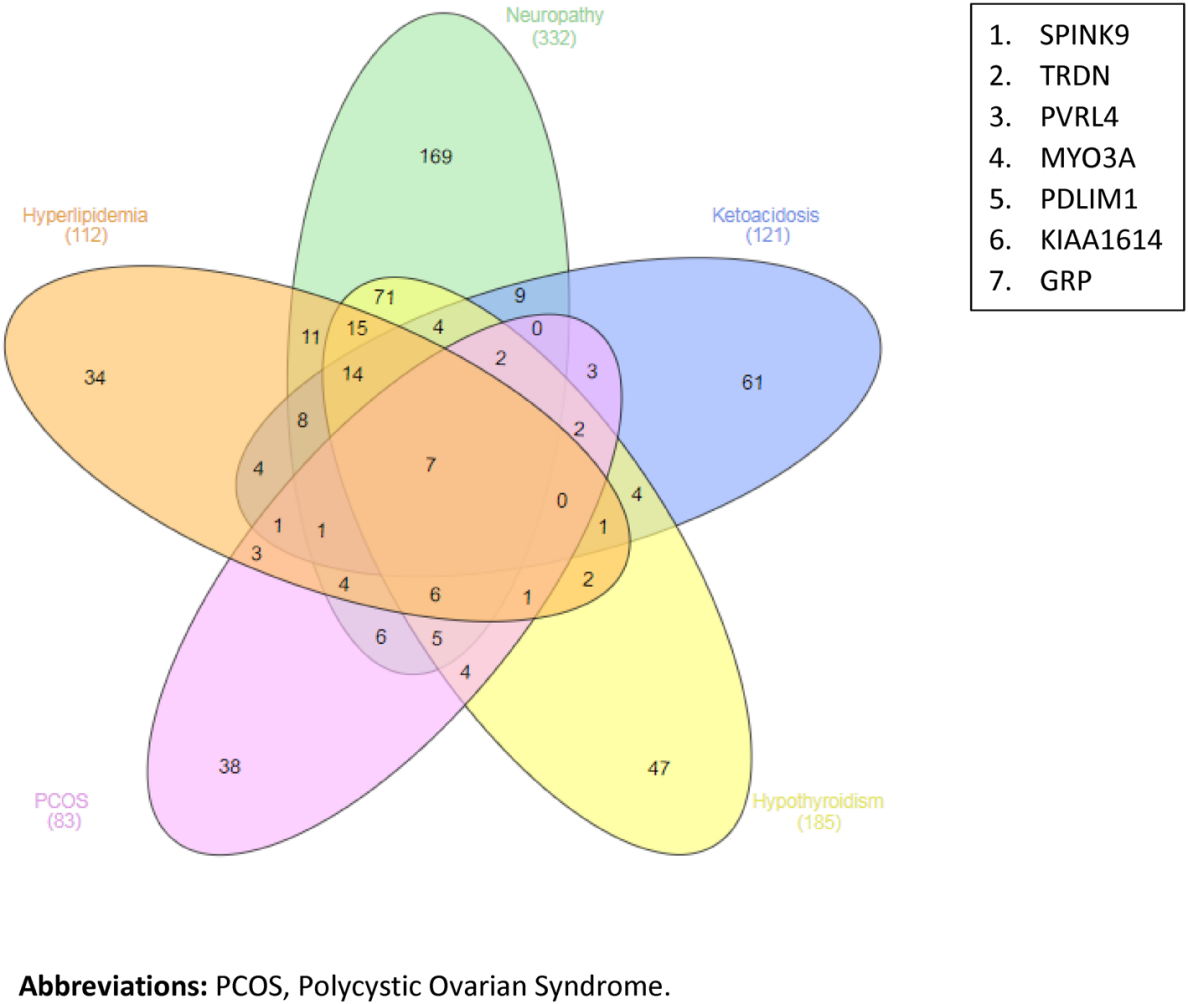
Differentially expressed genes common between all complications. A. Upregulated common genes; B. List of the seven upregulated common genes.

Analysis of upregulated DEGs has shown that the following seven genes were common between all complications of TIDM (hyperlipidemia, neuropathy, ketoacidosis, hypothyroidism and PCOS): *SPINK9, TRDN, PVRL4, MYO3A, PDLIM1, KIAA1614* and *GRP* (Fig. 6).

### Analysis of fold change of upregulated DEGs

Assessment of the relative fold-change of the seven upregulated genes in different T1DM complications showed a significant increase in expression of *SPINK9* in T1DM complications compared to DWC, hyperlipidemia (5.28), neuropathy (3.79), ketoacidosis (5.20), hypothyroidism (3.79) and PCOS (5.20) (Table 4). In addition, a significant increase in expression of *MYO3A* in hyperlipidemia (4.14), neuropathy (6.11), ketoacidosis (2.60), hypothyroidism (4.33) and PCOS (4.49) was observed.

**Table 4:** Relative fold changes of upregulated genes in different patients’ groups with T1DM complications compared to healthy control group.

Upregulated gene name	Patient group					
	No complication	Hyperlipidaemia	Neuropathy	Ketoacidosis	Hypothyroidism	PCOS
SPINK9^a^	1.30	5.28	3.79	5.20	3.79	5.20
TRDN	8.15	4.23	3.47	10.92	6.07	5.72
PVRL4	0.71	1.92	1.77	2.13	1.18	1.18
MYO3A^a^	1.73	4.14	6.11	2.60	4.33	4.49
PDLIM1	3.90	2.36	3.15	2.60	2.36	1.65
KIAA1614	1.81	2.36	2.17	1.89	3.74	2.36
GRP	2.06	1.63	5.69	4.23	3.25	4.88

^a^Indicates significant increases in the expression of the upregulated genes in different T1DM complications compared to no complications.

*PCOS* Polycystic Ovarian Syndrome.

### Prediction of onset of T1DM complications using ROC

**Figure 7:**
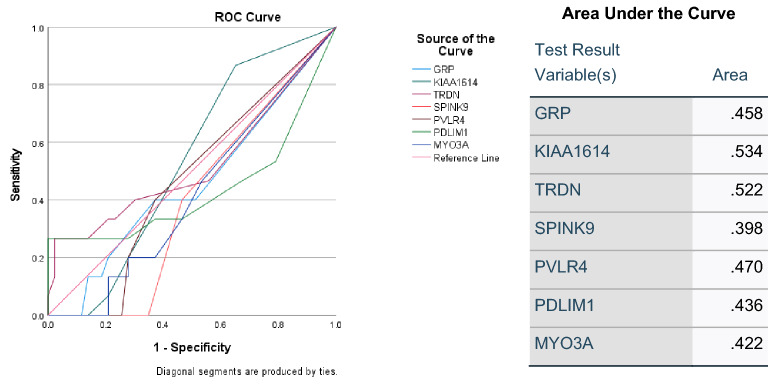
ROC curves of common upregulated DEGs between all T1DM complications. (A) ROC curves of upregulated common genes. (B) Area under the ROC curve. *ROC* receiver operating characteristic.

**Figure 8:**
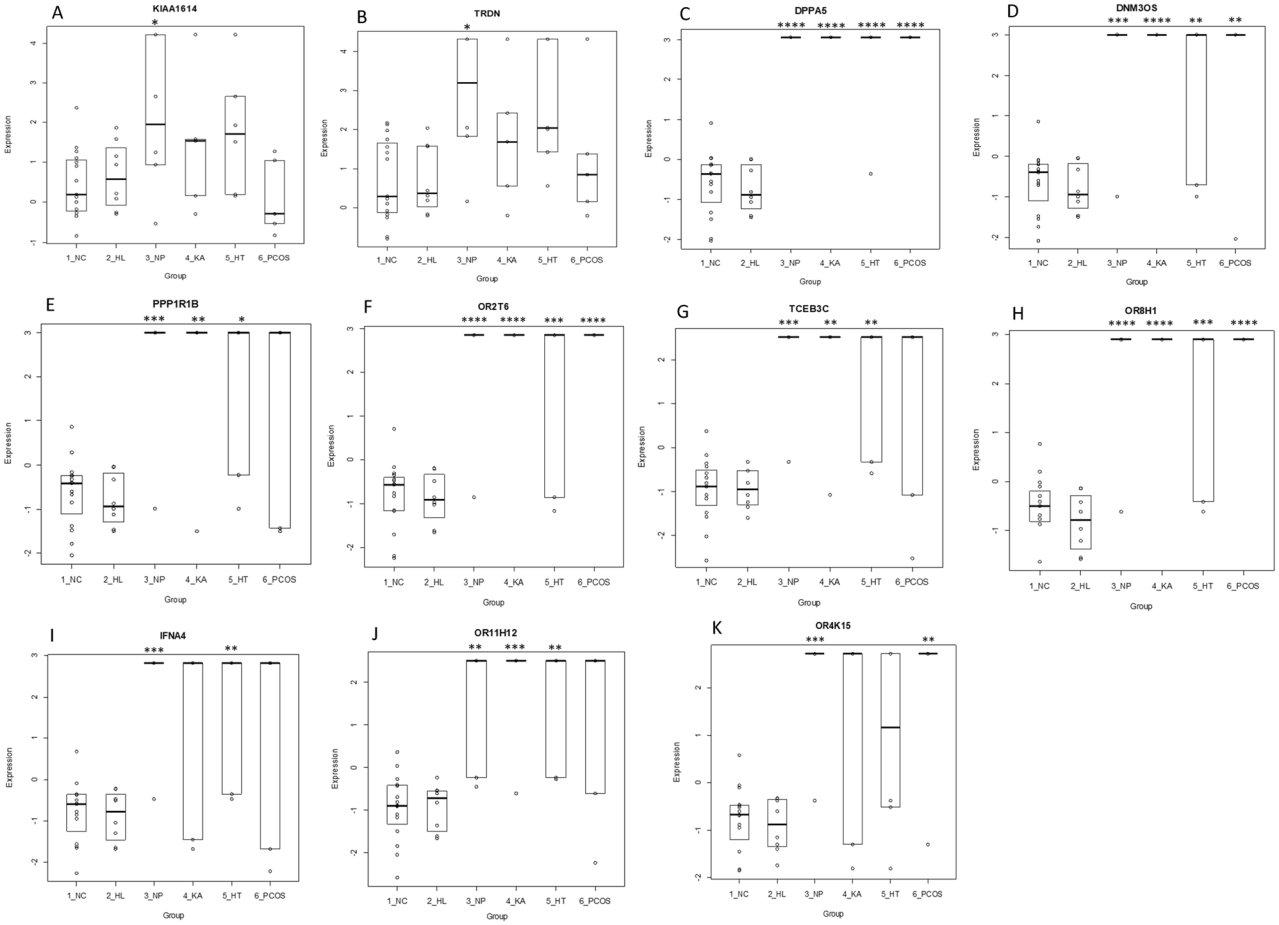
Boxplots of upregulated and downregulated genes differentially expressed between DWC and T1DM with complications. Fig. (**A,B**) show corresponding boxplots for the upregulated genes (*KIAA1614*, *TRDN*) whereas Fig. (**C**–**K**) show corresponding boxplots for the downregulated genes (*DPPA5*, *DNM3OS*, *PPP1R1B*, *OR2T6*, *TCEB3C*, *OR8H1*, *IFNA4*, *OR11H12*, *OR4K15*). Significance was calculated using one-way ANOVA between the DWC group versus the complication groups. P < 0.05 was considered significant (*P < 0.05; **P < 0.01, ***P < 0.001, ****P < 0.0001). *DWC* T1DM without complication, *HL* Hyperlipidemia, *NP* Neuropathy, *HT* Hypothyroidism, *KA* Ketoacidosis, *PCOS* Polycystic Ovarian Syndrome.

Receiver operating characteristic (ROC) analysis was used to identify predictive trend for the onset of T1DM complications. As shown in Fig. 7, among the common upregulated genes, *KIAA1614* and *TRDN* exhibited higher predictive value of 53% and 52%, respectively. The expression of these DEGs was investigated across all complications of T1DM including in hyperlipidemia, neuropathy, ketoacidosis, hypothyroidism and PCOS (Fig. 8). As shown in Boxplots 8A and 8B, *KIAA1614* and *TRDN* were significantly upregulated in neuropathy (p < 0.05). In addition, significant upregulation was observed in *DPPA5*, *DNM3OS*, *OR2T6*, *OR8H1* in four complications including neuropathy, ketoacidosis, hyperlipidemia and PCOs (p < 0.01) (Boxplots 8C, 8D, 8F,8H, respectively). Moreover, neuropathy, ketoacidosis and hyperlipidemia were associated with significant upregulations in expression of *PPP1R1B, TCEB3C* and *OR11H12* as shown in Boxplots 8E, 8G and 8J, respectively. On the other hand, upregulation of* IFNA4* was only observed in neuropathy and hypothyroidism and expression of *OR4k15* was increased significantly in neuropathy and PCOS as shown by Boxplots 8I and 8K, respectively (P < 0.01).

## Discussion

The incidence of diabetes has increased dramatically, and the complications that are associated with this chronic disorder represent a major global health challenge and socioeconomic burden^20^. Therefore, it is crucial to elucidate the genetic susceptibility for development of T1DM complications and identify the DEGs that are involved in the complex pathways of these complications. One of the significant outcomes of the present study is the identification of functional pathways that are involved in regulation of candidate genes that are responsible for the onset and development of T1DM complications. Regulation of ion transport pathway was identified as one of the top five pathways in neuropathy and this was supported by other studies which revealed that cellular and mitochondrial ion transport play a determinant role in pathogenesis of metabolic disorders including diabetes^21^. Further studies provided evidence for the association between the disturbed ion transport and neuropathy and highlighted ion channels as potential therapeutic target for neuropathy^22^. Interestingly, mineral absorption pathway was reported as one of the top pathways for regulation of the DEGs in DKA. It is documented that intact mineral absorption is prerequisite for metabolic homeostasis and mineral imbalance affects glucose metabolism and insulin sensitivity, adversely^23^. It is noteworthy that calcium ion transport pathway was associated with regulation of DEGs in PCOS and this highlighted the importance of calcium imbalance in ovarian pathologies^24^. It is evident that 50% of PCOS females have severe metabolic disorders including diabetes and recent studies have revealed an association between DEGs and activation of calcium ion binding in obese females with PCOS^25^.

The present study has, for the first time, identified the common upregulated DEGs between T1DM without complications and T1DM with multiple complications in UAE patients highlighting the candidate genes that may contribute to onset or development of complications in T1DM.

It is noteworthy that out of the seven upregulated DEGs, expression of *SPINK9* (Serine protease inhibitor Kazal type 9) and *MYO3A* (Myosin IIIA) were increased significantly in all T1DM complications compared to TIDM without complication and this highlights an important observation and introduces these two candidate genes as potential biomarkers for developing single or multiple complications in T1DM. Interestingly, previous investigations of Beta pancreatic cells using isolated PPARβ/δ-deficient islet, have demonstrated an association between dysfunctional insulin secretion and abnormal beta cell mass, and upregulation of *MYO3A* and *SPINK9*^26^. The latter was originally identified in human skin, particularly in the palmar epidermis, however, more recent studies have shown that *SPINK9* is also expressed in the pancreas^27^. Genetic mutations in other types of *SPINK* such as *SPINK1*, which is known as a pancreatic secretory trypsin inhibitor, are associated with chronic pancreatitis emphasizing that regulation of pancreatic proteases is an important factor for prevention of pancreatic autodigestion^28^. Altered expression of *SPINK9* in TIDM complications suggests the involvement of serine protease inhibitors in the pathophysiology of T1DM^29^. It is noteworthy that *SPINK9* was considered as predictor of type 2 diabetes mellitus (T2DM) features including insulin resistance and dyslipidemia^29^. In addition, interesting findings have suggested the involvement of *SPINK9* in different types of motor neuropathy using clinical presentation and measurement of conduction velocities^30^. Furthermore, several case reports highlighted a correlation between proteose inhibitors therapy and development of diabetic acidosis HIV-infected patients. Although the mechanisms underlying this correlation is unknown, it is postulated that protease inhibitors participate in the peripheral insulin resistance and glucose intolerance^31^. More importantly, previous investigations have confirmed that uncontrolled serine protease activity leads to profound immune responses and release of proinflammatory mediators^32^. This was supported by the association between abnormal serine protease activities and manifestations such as hypothyroidism and high serum IgE levels^32^. These immunometabolism features have also been used to explain the interrelationship between the alterations in skeletal and connective tissues that are associated with some of the diabetic complications including PCOS^33^. This may also provide an explanation for the increased expression of *SPINK9* in PCOS as shown in the present study.

On the other hand, *MYO3A* (Myosin IIIA) is one of the two genes encoding class III myosin which has isoforms A and B and is mainly expressed in the retina and cochlear hair cells^34,35^. Therefore, *MYO3A* mutations are strongly associated with altered photoresponse and survival, and with deafness, autosomal recessive and nonsyndromic hearing loss^34^. Given the significant involvement of the nervous system in the latter, it is plausible to suggest that *MYO3A* mutations may lead to neuropathy. This further supports our present finding that expression of *MYO3A* was significantly increased in T1DM patients with neuropathy. Interestingly, this increased expression of *MYO3A* was also observed in T1DM patients with hyperlipidemia and previous studies have demonstrated a correlation between abnormal lipid profile such as hypercholesterolemia and single nucleotide polymorphism in *MYO3A* which eventually affects the cardiac system, adversely^36^. It is noteworthy that an association was established between the dysfunction of the latter and DKA given the common pathophysiological mechanisms between the disorders which includes oxidative stress and systemic inflammation^37^.

On the other hand, increased expression of other five genes, *TRDN, PVRL4, PDLIM1, KIAA1614* and *GRP*, has been reported however, this increased fold was not statistically significant. *TRDN* (Triadin) was first identified to encode an integral membrane protein as a member of the muscle calcium release complex^38^. Although no previous research has been conducted to investigate the role TRDN in diabetes or glucose homeostasis, a recent case-report has speculated a relationship between genetic aberrations in *TRDN* and glucose-6-phosphate dehydrogenase deficiency indicating its putative role in complications of T1DM^39^. PVRL4 (Poliovirus receptor-like 4) gene encodes Nectin-4, a transmembrane glycoprotein and immunoglobulin-like cell adhesion molecule, which is involved in vital cellular processes including polarity, proliferation and differentiation^40^. Compared to other nectins, *Nectin-4* expression dominates during fetal development and early life and this is followed by reduction in *Nectin-4* expression with an expectation of abnormalities related to the development of tumor in several tissues including the pancreas^41^. Upregulation of *PVRL4* expression in T1DM patients with multiple complications as shown in the present study proposes an involvement of this gene in the pathophysiology of diabetes but further validation is warranted. PDLIM1 (Human PDZ and LIM domain protein 1), also known as CLP36, is a cytoplasmic LIM protein which is involved in negative regulation of NF-κB-mediated signaling in dendritic cells^42^. A distinctive expression profile of *PDLIM1* which is critically involved in NF-κB-mediated inflammation has been observed in the blood of patients with several chronic diseases including cardiovascular disease, hypertension, dyslipidemia and T2DM^43^. The latter can be used to hypothesize that the increased expression of *PDLIM1* in the present study is involved in development of T1DM complications including hyperlipidemia.

A genome-wide DNA methylation study which investigated newly hypermethylated genes in ulcerative colitis (UC) has demonstrated that *KIAA1614* significantly increased promoter methylation levels in UC compared to healthy control^44^. In addition, genome-wide DNA methylation analysis was used to identify the differentially methylated regions as novel potential epigenetic targets of metformin which is the main antihyperglycemic medication. The analysis has shown that *KIAA1614* was one of the main genes with the most consistent changes in the DNA methylation profile which suggests its involvement in energy homeostasis which is one of the main therapeutic targets of metformin^45^. Given that the main pathological feature underlying T1DM is autoimmune mechanisms, the increased expression of *KIAA1614* can reflects the involvement of these mechanisms in development of multiple complications. *GRP* (Gastrin-releasing peptide) is strongly involved in gastrointestinal inflammatory and metabolic diseases including diabetes^46^. Emerging evidence in pre-clinical and clinical studies has demonstrated that *GRP* is a key factor in regulation of glucose homeostasis in diabetes^46^. This was further supported by the finding that elevated levels of *GRP* were significantly associated with abnormal glucose metabolism after pancreatitis and increased levels of pro-inflammatory cytokines^46^. In agreement with these findings, the present study has shown that *GRP* was one of the top upregulated genes in patients with T1DM complications. Correlations between the latter and *GRP* were also observed in animal studies which investigated *GRP* Immunoreactivity in gastrointestinal tract of alloxan induced diabetes^47^.

On the other hand, the present study suggested an association between downregulated DEGs such as *DNM3OS* in development of T1DM, skeletal dysplasia and abnormal fat development^48^. Interestingly, the role of *DNM3OS* has also been reported in the inflammation that is associated with diabetes wherein abnormal expression of *DNM3OS* modulates the inflammatory function of macrophages in diabetes^49^. In addition, involvement of *TCEB3C* in the pathophysiology of T1DM has been suggested, previously^50^. This was highlighted by investigating the potential causative genes that increase the risk of developing T1DM and differential expression of *TCEB3* was noted in DNA microarray analysis in islet-specific CD4 + T cells of T1DM-susceptible NOD mice ^50^.

### Limitations of the study

One of the main limitations of the study is the sample size, although it is a multicenter study and different sites have been included, the number of patients with T1DM was small and this affected the number of patients in each complication group, adversely. Larger sample size will allow more thorough investigation of the combined complications as the present study evaluated each complication separately.

## Conclusion

The present study is the first to provide transcriptomic analysis for the common DEGs in Emirati patients with T1DM complications. Several functional pathways that regulate these DEGs were described including ion transport, mineral absorption and cytosolic calcium concentrations. In addition, upregulated DEGs that were common among all T1DM complications were identified. This included *SPINK9*, *TRDN*, *PVRL4*, *MYO3A*, *PDLIM1*, *KIAA1614* and *GRP*. The findings of the present study support the potentiality of *SPINK9* and *MYO3A* as candidate biomarkers for development of T1DM complications. The study provides a reference for further in-depth studies to validate the involvement of these DEGs in onset and development of T1DM complications.

## Supplementary Information

Supplementary Information 1.

Supplementary Figures.

Supplementary Tables.

Supplementary Tables.
