# Supplementary material for: Identification of novel differentially expressed genes in type 1 diabetes mellitus complications using transcriptomic profiling of UAE patients: a multicenter study
Source: Sci Rep. 2022 Sep 29;12:16316. doi: 10.1038/s41598-022-18997-w (PMC9523055; doi:10.1038/s41598-022-18997-w)
Supplement: Supplementary file 2 — Supplementary Figures. [file 41598_2022_18997_MOESM2_ESM.docx]

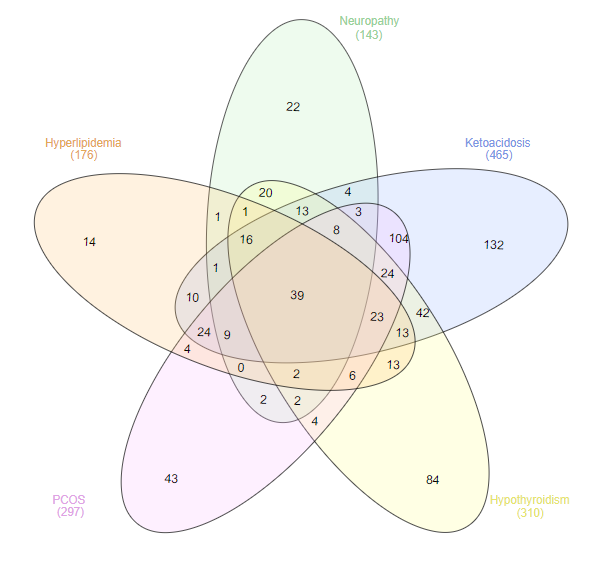
Figure 1S : Common downregulated shared genes between all T1DM complication

21. VN1R4
 22. OR2T6
 23. PPP1R1B
 24. OR5P2
 25. OR4M2
 26. OR4F21
 27. LOC339240
 28. OR6C75
 29. DNM3OS
 30. OR52A5
 31. USP17L6P
 32. LOC732275
 33. OR52A1
 34. RYR1
 35. GOLGA6C
 36. DPPA5
 37. USP17L5
 38. KRT16P3
 39. LOC349196

1. C3orf74
2. PRR19
3. STRC
4. KRT16P2
5. OR4K15
6. OR4C46
7. OR11H12
8. DUOX2
9. SLC38A3
10. OR4C13
11. IFNA4
12. TEAD4
13. OR4K14
14. LOC100506888
15. RDH8
16. PURG
17. LOC389791
18. TCEB3C
19. REXO1L2P
20. OR8H1

Figure 1S. Common downregulated differentially expressed genes between all T1DM complications. Common 39 downregulated DEGs and list of the common 39 DEGs. Abbreviations: DEGs, differentially expressed genes.

| **Area Under the Curve** | |
| --- | --- |
| Test Result Variable(s) | Area |
| DPPA5 | .651 |
| DNM3OS | .647 |
| PPP1R1B | .660 |
| OR2T6 | .630 |
| TCEB3C | .621 |
| OR8H1 | .620 |
| IFNA4 | .612 |
| OR11H12 | .618 |
| OR4K15 | .626 |

Figure 2S: ROC analysis for downregulated genes


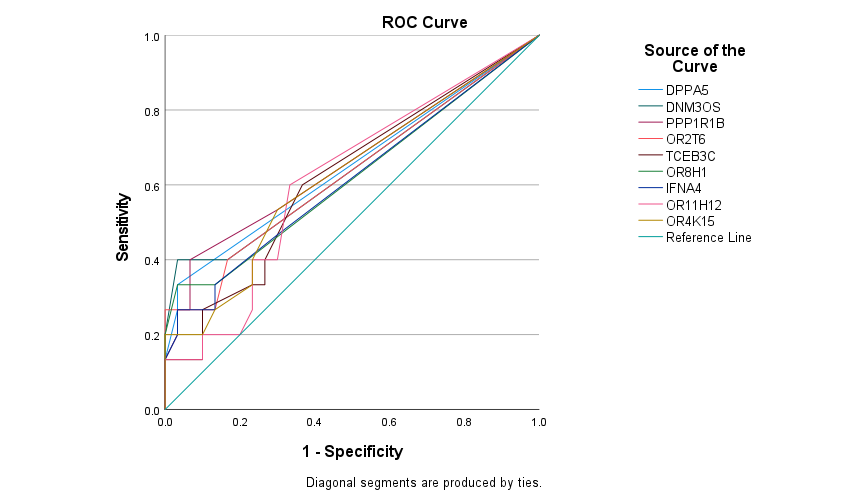


Figure 2S. ROC curves of downregulated common DEGs between all T1DM complications. Abbreviations: AUC = area under the ROC curve; ROC = receiver operating characteristic.
